# Supplementary material for: Cholesterol Levels Affect the Performance of AuNPs-Decorated Thermo-Sensitive Liposomes as Nanocarriers for Controlled Doxorubicin Delivery
Source: Pharmaceutics. 2021 Jun 27;13(7):973. doi: 10.3390/pharmaceutics13070973 (PMC8309145; doi:10.3390/pharmaceutics13070973)
Supplement: Supplementary file 1 [file pharmaceutics-13-00973-s001.zip › pharmaceutics-1259736-supplementary.pdf]

pharmaceutics

MDPI

Supplementary Materials: Cholesterol Levels Affect the Performance of AuNPs-Decorated Thermo-Sensitive Liposomes as Nanocarriers for Controlled Doxorubicin Delivery

Mónica C. García, Nabila Naitlho, José Manuel Calderón-Montaño, Estrella Drago, Manuela Rueda, Marcela Longhi, Antonio M. Rabasco, Miguel López-Lázaro, Francisco Prieto-Dapena and María Luisa González-Rodríguez

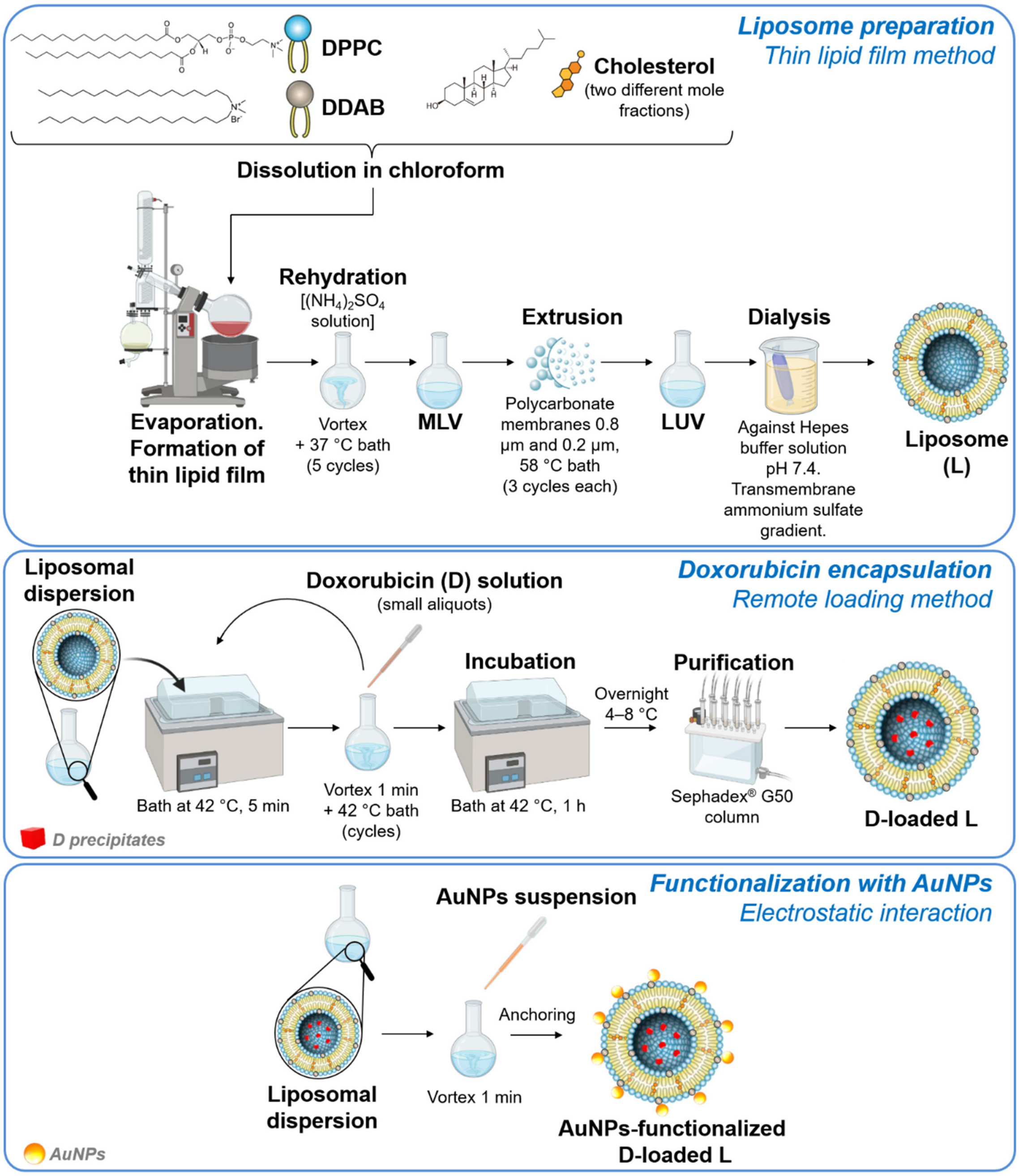

Figure S1. Schematic depiction of liposome (L) preparation, doxorubicin (D) encapsulation and anchoring process with gold nanoparticles (AuNPs).

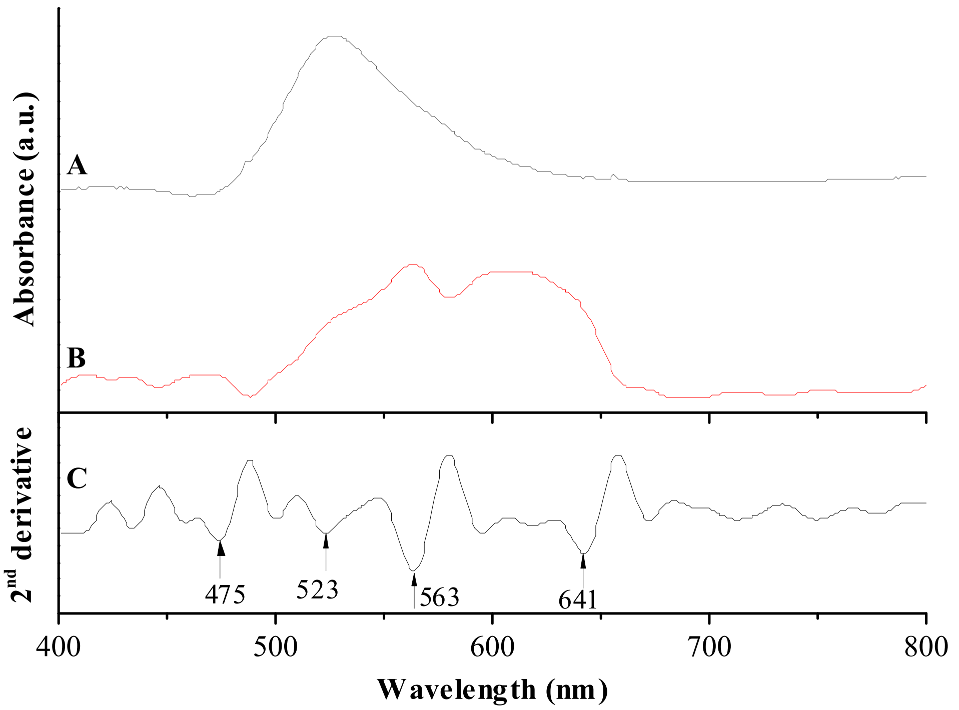

Figure S2. Absorption UV-Visible spectra of **A)** gold nanoparticles (AuNPs), **B)** Doxorubicin-loaded AuNPs-anchored liposomes with high proportion of cholesterol (AuNPs-L2-D), and **C)** second derivative of the spectra in **B)**, pointing the minima that represent the positions of absorption bands convoluted in the original spectra.

The absorption bands at 475 nm in the spectra (Figure S2B) can be assigned to the absorption of D- loaded L2. The band at 523 nm corrresponds to the surface plasmon of independent AuNPs, and has the same wavelength that the main band in AuNPs spectra (Figure S2A). Bands at 563 and 641 nm must correspond to the surface plasmon of AuNPs partially aggregated.

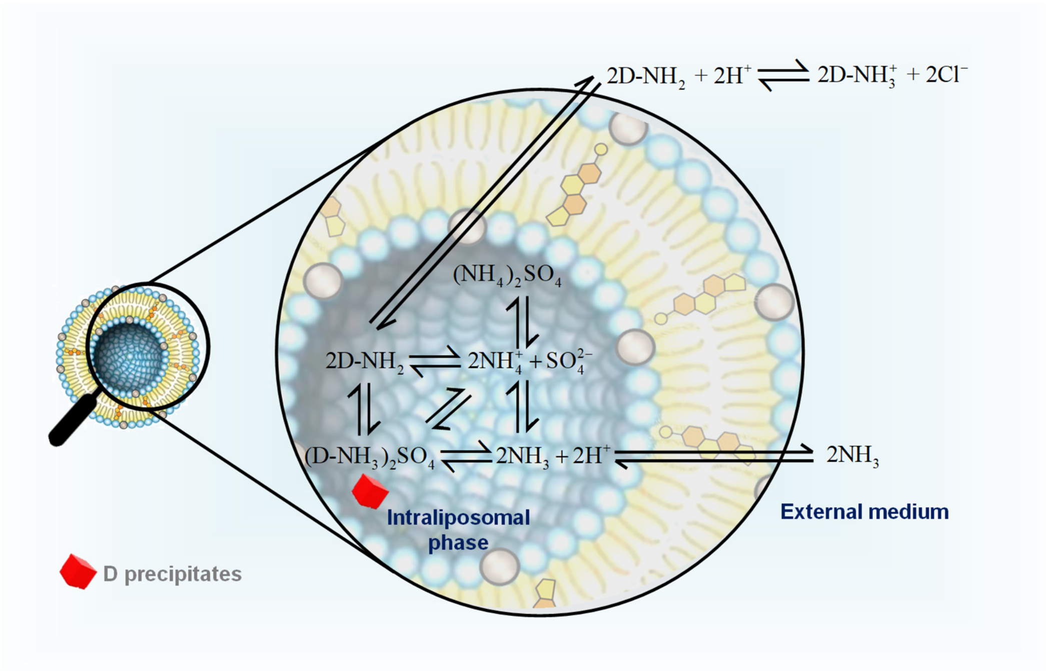

Figure S3. Mechanism of active loading of doxorubicin (D) into the intraliposome aqueous phase by transmembrane ammonium sulfate gradient.
